# Glucose Reduces Norovirus Binding to *Enterobacter cloacae* and Alters Gene Expression of Bacterial Surface Structures in a Growth Phase Dependent Manner

**DOI:** 10.3390/v14081596

**Published:** 2022-07-22

**Authors:** Kendall J. Long, Chanel A. Mosby, Melissa K. Jones

**Affiliations:** Microbiology and Cell Science Department, IFAS, University of Florida, Gainesville, FL 32611, USA; kendall.long@ufl.edu (K.J.L.); c.mosby.haundrup@ufl.edu (C.A.M.)

**Keywords:** norovirus, murine norovirus, human norovirus, virus-bacterial interaction, gene expression

## Abstract

Norovirus is the leading cause of acute viral gastroenteritis. Both human and murine noroviruses attach to commensal bacteria belonging to the mammalian gut flora, and binding levels are influenced by nutrients present in bacterial media. However, it is not known which nutrients are responsible for altering viral binding or why binding is altered. Gene expression of commensal bacteria can be changed by the external environment as well as by interaction with pathogens. For example, growth phase and incubation conditions impact expression levels of specific bacterial genes in *Escherichia coli*. We have previously shown that binding by both human and murine noroviruses to the commensal bacterium *Enterobacter cloacae* induces genome-wide changes in gene expression with a large number of differentially expressed genes associated with the surface structure of the bacterial cell. The current study evaluated norovirus binding under nutrient-limited conditions and assessed the expression of a select panel of these genes that are significantly altered by norovirus binding under these conditions. The goal of this work was to determine how norovirus attachment to *Enterobacter cloacae* affected the expression of these genes under varying nutrient and growth phase conditions. We found that the presence of glucose in minimal media reduced murine norovirus binding to *E. cloacae* and viral binding in the presence of glucose reduced gene expression for surface structures previously associated with norovirus attachment. Changes in viral binding and gene expression occurred in a growth phase-dependent manner. Collectively, these data demonstrate that both the growth phase and nutrient availability alter viral interactions with commensal bacteria and the subsequent changes in gene expression. Ultimately, this work advances our understanding of norovirus-bacterium interactions and provides a foundation for elucidating the conditions and surface structures that regulate norovirus attachment to bacteria.

## 1. Introduction

Human norovirus (HNoV) is the leading cause of acute gastroenteritis in the United States for people of all ages [1,2]. Infection by HNoV most commonly occurs when infected feces, food, or fluid are ingested [3], revealing the highly contagious nature of the virus, which can lead to life-threatening situations or prolonged infection for young, elderly, and immunocompromised individuals [4]. Despite the impact of this disease, there is still no approved antiviral drug or vaccine available for noroviruses [2].

Glycans play an important role in norovirus infection. Previous studies of HNoVs have revealed that histo-blood group antigens (HBGAs), which are present on the surface of red blood cells and mucosal epithelial cells, are likely important for viral interaction with the host [5,6] and may increase viral infection rates within the body [1,7]. Carbohydrates similar to HBGAs are also found on the surface of bacterial cells [8], potentially facilitating interaction between HNoV and commensal bacteria. Indeed, several groups have demonstrated that HNoVs effectively bind a wide array of bacterial species with high efficiency [1,9,10]. For some bacterial species, this interaction with HNoV has been linked to the expression of HBGA-like compounds on the bacterial surface [9]. Specifically, HNoV has been shown to interact with the HBGA-like compounds on the surface of the commensal proteobacterium *Enterobacter cloacae* and the presence of this bacterium enhances HNoV infection of B cells, further revealing the important role certain bacterial species play in HNoV infection efficiency [7,9]. In addition to binding HBGA-like compounds, electron microscopy has revealed that HNoV can also bind to other bacterial surface structures, including pili, flagella, and the outer membrane [1]. Like HNoV, murine norovirus (MNV) also binds to several commensal bacteria belonging to phyla prominent in the intestinal microbiome [2] and the presence of commensal bacteria enhances MNV replication in vivo [7,11].

Not only do commensal bacteria alter norovirus infection, but norovirus interactions with these bacteria induce changes in bacterial gene expression and physiology [12]. Specifically, norovirus interactions with *E. cloacae* significantly alter the expression of genes associated with the bacterial membrane, leading to increased expression of bacterial appendages on the cell surface and increased membrane ruffling. Changes in the external environment, as well as changes in the bacterial growth phase and incubation conditions, have specific impacts on bacterial gene expression and phenotypes for many well-studied bacterial species [13,14,15]. For example, incubation in minimal media reduces biofilm formation and adhesion abilities of *E. coli* and other bacterial species [14,16]. It has also been found that specific components of minimal media, such as glucose, may be directly responsible for specific reductions in gene expression within *E. coli*, including flagellar synthesis [17]. Glucose concentrations within the gut are known to shift based on a variety of factors such as host nutrient intake [18] as well as gut microbiota composition [19]. Understanding how norovirus binding responds to these specific changes in glucose concentration can therefore allow for a better understanding of why norovirus infectivity may be enhanced within certain host organisms. Changes in growth medium and conditions can also influence the efficiency of norovirus binding to commensal bacteria where growth in enriched media significantly reduced viral binding [1], but the components within these media that reduced binding were not determined.

Based on this data and the phenotypic changes induced by norovirus interactions, we examined how changes in the bacterial growth phase and nutrient parameters impacted viral binding to *E. cloacae* and the expression of specific genes associated with bacterial surface structures. We found that the presence of glucose reduced norovirus binding as well as expression of flagellar and outer membrane genes that are typically upregulated upon norovirus interactions. Gene expression in the presence of norovirus also differed between log and stationary phase *E. cloacae*. Collectively, these data demonstrate that specific nutrients, as well as the bacterial growth phase, can alter norovirus interactions with commensal bacteria.

## 2. Materials and Methods

### 2.1. Bacteria Strains and Virus Production

*Enterobacter cloacae* (ATCC 13047) was used to perform the norovirus-bacteria attachment assays. For all stationary phase conditions, *E. cloacae* cultures were grown overnight at 37 °C with constant agitation (200 rpm) in Luria Bertani (LB) media until cultures reached an OD_600_ of 1.7 to 1.9. For mid-logarithmic (mid-log) phase conditions, bacterial cultures were incubated at 37 °C in LB media with constant shaking (200 rpm) until an OD_600_ of 0.55 to 0.65. MNV-1 recombinant virus stocks were generated using pSPMNV-1.CW3 (generously provided by Dr. Stephanie Karst) as previously described and stored at −80 °C [20]. HNoV virus-like particles (VLPs) were purchased from Creative Biosciences (#CBS-V700) and stored at −80 °C [20].

### 2.2. Norovirus-Bacteria Attachment Assay

Attachment assays were performed as previously described [20]. Briefly, after growth to stationary or mid-log phase, bacterial cultures were washed twice with 1X PBS. The culture concentration was then adjusted to 3 × 10^8^ CFU/mL using the appropriate diluent (i.e., PBS or M9 minimal media), and 1 mL aliquots were transferred into a 1.5 mL microcentrifuge. The M9 minimal media had a final glucose concentration of 0.4%. For the PBS with 2% glucose (PBS-G) diluent condition, we adjusted the concentration of bacteria to 3 × 10^8^ CFU/mL using PBS as the initial diluent, then transferred 1 mL of culture into 2.0 mL microcentrifuge tubes, followed by the addition of either 0.25 mL, 0.5 mL, or 1 mL of 2% glucose for a final concentration of 0.3%, 0.6%, or 1.2% glucose, respectively. For each combination of the growth phase and media, bacterial cells were inoculated with either HNoV VLPs (0.1 µg/mL), MNV (0.1 MOI), or mock inoculated with PBS (no virus). All tubes were then incubated at 37 °C with gentle mixing for 1 h. Technical replicates (*n* = 3) were included for each condition in each experiment, and each experiment was performed three times.

### 2.3. RNA Extraction and DNase Treatment

RNA extraction and DNase treatment were performed to measure both levels of bacterial gene expression and MNV attachment. After incubation, RNA*later* (Ambion, Austin, TX, USA) was added to each tube. RNA extraction was performed using the Zymo Quick-RNA^TM^ Miniprep Kit (Zymo Research, Irvine, CA, USA) per the manufacturer’s instructions but included an additional wash step prior to the dry spin before RNA elution. RNA concentrations were measured using a Nanodrop (Thermo Fisher, Waltham, MA, USA) and remaining gDNA was removed using the Turbo DNA-free kit (Ambion, Austin, TX, USA) per the manufacturer’s instructions. The DNase-treated RNA was then stored at −80 °C.

### 2.4. RT-qPCR

The M-MLV Reverse Transcriptase kit (Promega, Madison, WI, USA) was used to generate cDNA the DNase-treated RNA, and RT-negative controls were used for all samples. PowerUp SYBR Green Master Mix (Applied Biosystems, Waltham, MA, USA) and 300 nM of primers (Table S1) were used to amplify the cDNA on a QuantStudio 3 qPCR system (Applied Biosystems, Waltham, MA, USA). Relative gene expression of our target genes was calculated using the 2^−ΔΔCq^ method by normalizing the target gene expression to an endogenous control, *rpoB*, then normalizing the samples to the mock-inoculated PBS control [21]. For MNV quantification, serial dilutions of pSPMNV-1.CW3 were included on all MNV amplification plates and used to generate a standard curve.

### 2.5. Statistical Analysis

**Figure 1:**
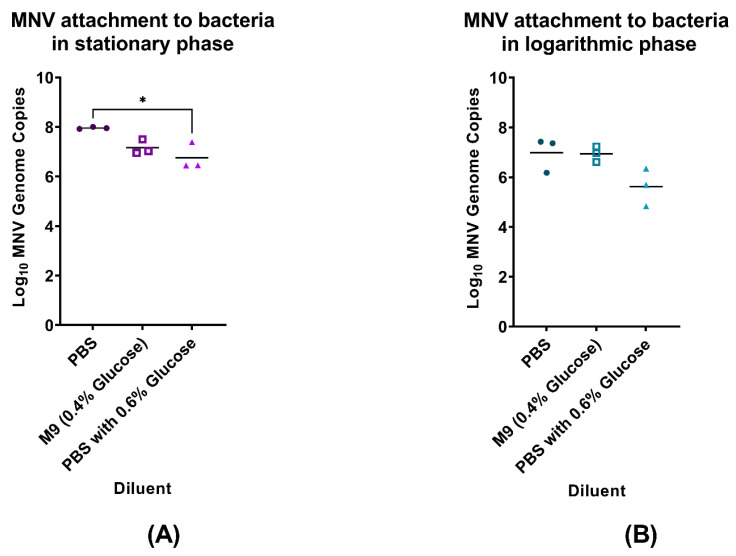
Impact of glucose and bacterial growth phase on MNV attachment to *E. cloacae.* MNV genome copies were measured using RT-qPCR after 1 h of viral attachment to *E. cloacae* in PBS, M9, or PBS-G media. The attachment was assessed using *E. cloacae* in (**A**) stationary and (**B**) mid-log growth phases. * designates an adjusted *p*-value of ≤0.05; *n* = 3.

**Figure 2:**
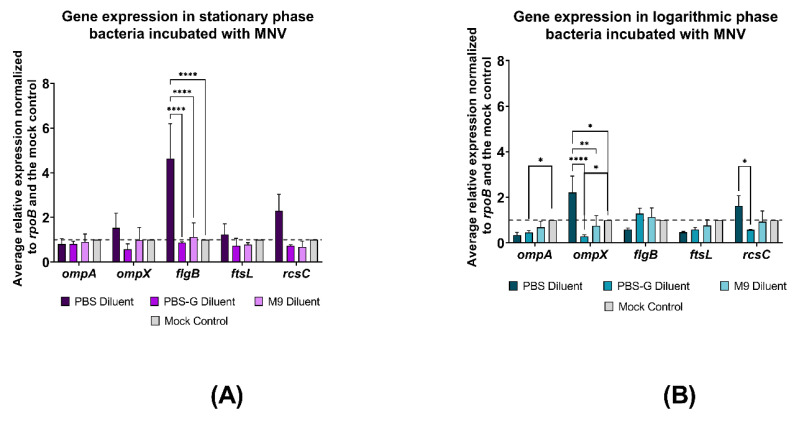
Growth phase results in the difference in gene expression when murine norovirus is attached to *E. cloacae*. (**A**) Differences in gene expression measured via qPCR when MNV interacts with *E. cloacae* at stationary growth phase; (**B**) differences in gene expression measured via qPCR when MNV interacts with *E. cloacae* at mid-log growth phase. Relative expression and significance are measured against gene expression of a mock control of *E. cloacae* without MNV, signified by a dashed line at y = 1 and the mock control column. * designates an adjusted *p*-value of ≤0.05, ** ≤ 0.01, *** ≤ 0.001, **** ≤ 0.0001 and the error bars show SEM; *n* = 3.

**Figure 3:**
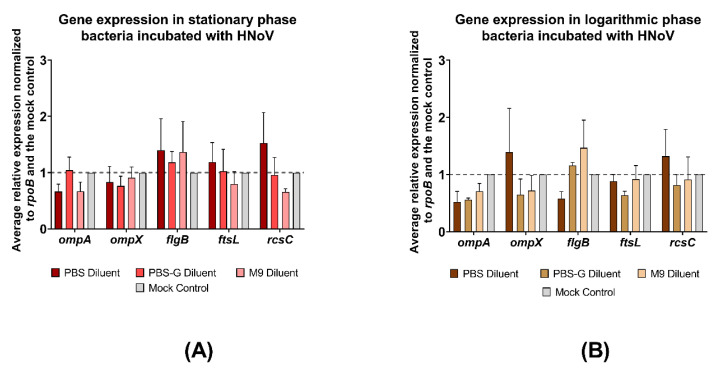
The bacterial growth phase and media do not significantly alter *E. cloacae* gene expression during HNoV binding. (**A**) Differences in gene expression measured via qPCR when HNoV interacts with *E. cloacae* at stationary growth phase; (**B**) differences in gene expression measured via qPCR when HNoV interacts with *E. cloacae* at mid-log growth phase. Relative expression and significance are measured against gene expression of a mock control of *E. Cloacae* without MNV, signified by a dashed line at y = 1 and the mock control column. Error bars show SEM; *n* = 3.

**Figure 4:**
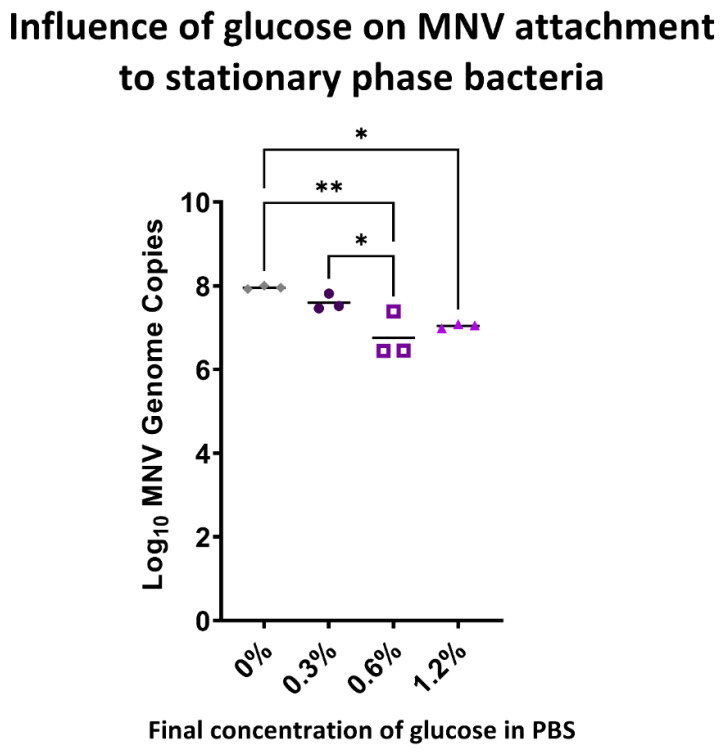
Impact of varying glucose concentrations on MNV attachment to *E. cloacae.* MNV genome copies were measured using RT-qPCR after 1 h of viral attachment to *E. cloacae* in PBS only (0% glucose) or PBS-G mediums with 0.3%, 0.6%, or 1.2% glucose. The attachment was assessed using *E. cloacae* in the stationary phase. * designates an adjusted *p*-value of ≤0.05, ** designates an adjusted *p*-value of ≤0.005; *n* = 3.

**Figure 5:**
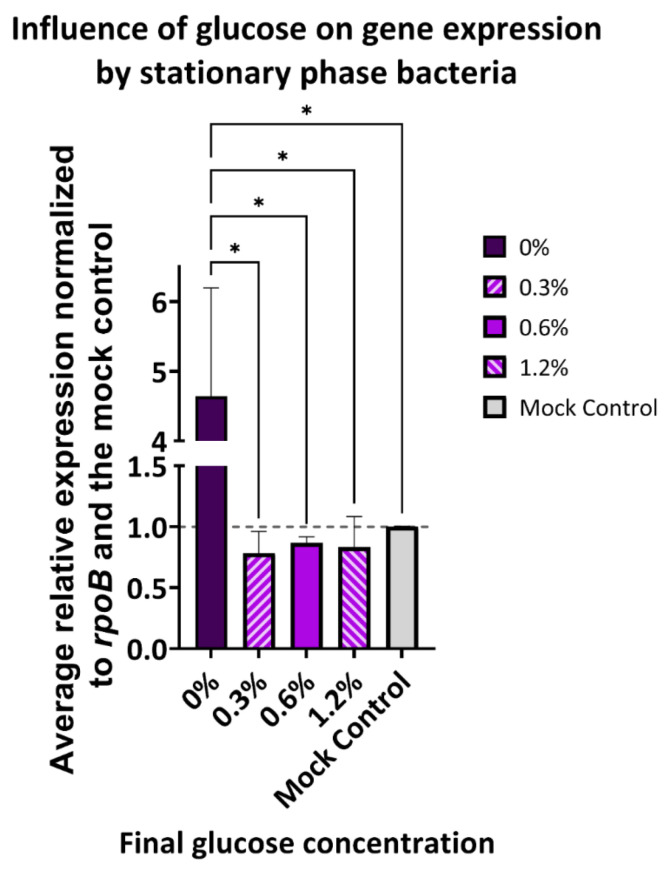
Varying glucose concentrations do not significantly alter *flgB* expression in *E. cloacae* following incubation with MNV. Differences in gene expression were measured via qPCR when MNV interacts with *E. cloacae* in the stationary growth phase. Relative expression and significance are measured against gene expression of a mock control of *E. Cloacae* without MNV, signified by a dashed line at y = 1 and the mock control column. (* = *p* ≤ 0.05). The error bars show SEM; *n* = 3.

Statistical significance for Figure 1 was determined by one-way ANOVA with Tukey’s multiple comparisons test. Statistical significance for Figure 2 and Figure 3 was determined using two-way ANOVA with Tukey’s multiple comparisons test. Statistical significance for Figure 4 and Figure 5 was determined by one-way ANOVA with Tukey’s multiple comparisons test. All figures and statistical analyses were made using GraphPad Prism (version 9.2.0, GraphPad Software, San Diego, CA USA, www.graphpad.com, last accessed on 8 July 2022). Statistical significance was calculated between an individual condition and its mock inoculated PBS control.

## 3. Results

### 3.1. Impact of Bacterial Growth Medium on MNV Binding to Enterobacter cloacae

Since it has been previously demonstrated that high nutrient media results in decreased norovirus binding compared to low nutrient media [1], we questioned if smaller changes in nutrient availability would alter viral interactions with bacteria. Thus, our studies quantified MNV attachment to *E. cloacae* using PBS and M9 minimal media. We compared MNV attachment in PBS versus M9 media, where PBS is a non-nutritive saline solution and M9 is a saline-based medium with added glucose, creating a 0.4% glucose solution. Results from these experiments showed that, although not significantly lower, when *E. cloacae* was in the stationary phase, levels of MNV attachment were decreased in M9 compared to PBS (Figure 1A). Additionally, although not statistically significant, during mid-log growth, the viral attachment was reduced in PBS compared to stationary growth samples and is at levels similar to those in the M9 medium condition (Figure 1B), which is different from what we previously observed [20]. Given that the primary difference between M9 and PBS is the presence of glucose, this indicated that glucose may be preventing interaction between the virus and bacterium. Furthermore, HNoVs are well known to interact with carbohydrate structures on the surface of bacterial cells [7,9]. Therefore, we wanted to determine if high glucose concentrations would impact MNV attachment to bacteria. We hypothesized that if glucose residues are involved in MNV attachment to bacteria, then if glucose was present in the medium at sufficiently high concentrations, it would interact with the virus and subsequently decrease MNV attachment to bacteria. We therefore chose to test MNV attachment levels in a higher glucose PBS-G (0.6% glucose) solution and found, although not significantly lower, decreased attachment levels when compared to attachment in M9 and significantly lower attachment levels when compared to attachment in PBS, indicating a potential correlation between glucose and MNV attachment to *E. cloacae.* These results showed that MNV attachment in PBS-G was significantly less compared to PBS and similar to attachment in M9 media when bacteria were in the stationary phase (Figure 1A). Interestingly, although not statistically significant, when bacteria were in mid-log phase growth, the addition of glucose to PBS not only decreased viral attachment compared to PBS alone but also reduced attachment to levels below what is observed in M9 media (Figure 1B). Together, these results are consistent with previous reports demonstrating that viral binding to bacteria is inversely related to the presence of nutrients in the medium and indicate that glucose may be partially responsible for this difference in attachment.

### 3.2. Effect of Bacterial Medium, Growth Phase, and MNV Attachment on E. cloacae Gene Expression

We have previously shown that norovirus interactions with *E. cloacae* induce genome-wide changes in bacterial gene expression that are linked to physiologic changes in the architecture of the bacterial surface. These physical changes include increased membrane ruffling, OMV productions, and the presence of surface appendages. Based on the impact of glucose on viral binding (Figure 1), we measured the expression of five structural genes that were previously shown to be differentially expressed by norovirus binding (Table 1). RT-qPCR was used to quantify gene expression, then Cq values were normalized to the internal control gene *rpoB* and then normalized again to expression of the target gene in *E. cloacae* that was not exposed to MNV. This allows for ease in comparing the fold change of the target gene in viral conditions to the expression of the target gene when virus is not present. Results showed that expression of *flgB* was significantly higher in PBS samples upon norovirus exposure, where expression of this gene did not change in M9 and PBS-G when *E. cloacae* was in the stationary phase (Figure 2A). In addition, *rscC* expression was also higher in PBS samples upon norovirus interaction, although this increase was not significant. The expression of the remaining genes was similar among the various media and was not significantly different from expression in mock-inoculated *E. cloacae* grown to stationary phase (Figure 2A). Since the bacterial growth phase altered viral binding, we also assessed gene expression under these conditions. When *E. cloacae* was in logarithmic growth during MNV attachment, gene expression patterns differed compared to stationary phase. Specifically, the expression of *flgB* was no longer upregulated in PBS samples (Figure 2). Differences in *ompA* and *ompX* expression were also observed during the logarithmic growth phase. Expression of both *ompA* and *ompX* was significantly reduced in the PBS-G condition upon norovirus binding. In addition, *ompX* was increased in PBS alone but not significantly (Figure 2B).

### 3.3. E. cloacae Gene Expression during HNoV-VLP Attachment Is Not Altered by Incubation Medium or Growth Phase

In addition to assessing gene expression during MNV attachment, we also evaluated expression during HNoV interactions during stationary and mid-log *E. cloacae* growth. While significant differences in gene expression were not observed, expression of all target genes except *flgB* were under-expressed in the M9 condition compared to the mock control during *E. cloacae* stationary phase growth (Figure 3A). In the presence of glucose, and specifically in the PBS-G samples, expression of *ompX* and *rcsC* were under-expressed, while *ompA*, *ftsL*, and *flgB* were slightly over-expressed (Figure 3A). When *E. cloacae* was in the mid-log phase growth, expression of all target genes except *flgB* were under-expressed in the M9 condition when compared to the mock control, although none of the changes were significant (Figure 3B). We saw similar results in the PBS-G media, where expression of all target genes except *flgB* were under-expressed (Figure 3B). Furthermore, the expression of *ompX* and *rcsC* were lower in media containing glucose compared to PBS alone (Figure 3B).

### 3.4. Stationary Phase E. cloacae MNV Attachment Levels Are Altered by Glucose While flgB Expression Following MNV Incubation Is Not

In our previous results, we found that the presence of glucose in M9 minimal media (0.4% glucose), as well as the PBS-G media (0.6% glucose), reduced MNV attachment levels as well as *flgB* expression in stationary phase *E. cloacae* incubated with MNV. To further determine if MNV attachment levels and *flgB* expression are influenced by glucose concentration, we examined MNV attachment and gene expression under additional glucose concentrations. Using stationary phase *E. cloacae* incubated with MNV in PBS-G media with final concentrations of 0.3% and 1.6% glucose, we found that MNV attachment levels in the 0.3% PBS-G condition are significantly increased when compared to attachment levels originally observed at 0.6% PBS-G condition (Figure 1A and Figure 4). Although not significant, we found that the 0.6% and 1.2% PBS-G condition have similar MNV attachment levels and that the 0.3% PBS-G condition has slightly higher levels of MNV attachment when compared to the M9 minimal media condition with a final concentration of 0.4% glucose (Figure 1A and Figure 4). We additionally found that *flgB* expression was not significantly impacted by increasing or decreasing the glucose concentration of the MNV incubation medium (Figure 5).

## 4. Discussion

To better characterize MNV binding to commensal bacteria levels of MNV attachment to *E. cloacae* were measured under varying nutrient and bacterial growth phase parameters. We found that MNV attachment to *E. cloacae* in stationary phase was significantly lower in the PBS-G and insignificantly lower in the M9 minimal media compared to the PBS diluent condition. Upon further testing, we found that MNV attachment levels are likely glucose concentration-dependent as reducing glucose levels from 0.6% to 0.3% significantly increased MNV attachment levels to stationary phase *E. cloacae*. This trend indicates that glucose may be hindering viral attachment to the bacterial cell. Lowered binding may be due to glucose directly blocking binding sites on the bacterium and/or viral surfaces, or it may be altering the expression of the bacterial surface structures to which the virus binds. Glucose can reduce expression levels of certain bacterial outer membrane proteins during both stationary and log phase [15,22]. Minimal media, specifically those containing glucose such as M9, have also been found to reduce expression levels of certain outer membrane proteins involved in biofilm formation, motility, and adhesion [14,23,24]. Furthermore, *Citrobacter freundii* grown in glucose had lower levels of membrane proteins and lipids [25]. Thus, the glucose in M9 and PBS-G media may be reducing the presence of certain potential MNV binding receptors found on the *E. cloacae* outer membrane, ultimately causing a decrease in MNV attachment to *E. cloacae*.

To further explore this hypothesis that glucose is reducing the expression of key bacterial surface structures, we measured the expression of genes associated with bacterial surface structures previously associated with norovirus interactions (e.g., the flagellum and the outer membrane) [1,20]. We have previously found that norovirus interactions with *E. cloacae* significantly alters the expression of genes associated with the bacterial outer and trans membranes [12]. We also know that MNV binding to bacteria is variable, with 10% to 20% of bacteria remaining unbound by MNV following incubation with the virus, which may impact the consistency of gene expression data found in each replicate [20]. Additional research on *E. coli* and *Shigella flexneri* has found that the expression of genes involved in cell growth is affected by the bacterial growth phase [13,26]. Therefore, we measured the expression of select genes chosen for their involvement in *E. cloacae* cell structure and growth as well as a flagellar gene since HNoV has been shown to bind to this appendage [1]. Overall, our results showed that both the growth phase and the presence of glucose altered gene expression of these genes following viral attachment to *E. cloacae*. This may imply that norovirus binding affects gene expression in *E. cloacae* through the activation of specific genes involved in cell growth.

Although stationary phase and mid-log phase experiments each had individual mock controls (no virus) in their respective growth phases, certain general statements can be made about the impact growth phase may have on gene expression in bacteria infected with MNV due to clear differences in up- or down-regulation as well as significance between specific genes and their mock controls in bacteria of different growth phases. When evaluating the impact of growth medium on gene expression during MNV attachment, we found that both stationary and mid-log phase *E. cloacae* had insignificant changes in gene expression between each target gene in M9 media and their respective controls, indicating that nutrient presence could negate changes in gene expression normally observed between growth phases. This trend could potentially be due to environmental stresses caused by M9 minimal media on *E. cloacae* [27,28,29], leading it to ultimately have a greater impact on bacterial gene expression than the impact of the growth phase [14,28,30,31,32]. Interestingly, *ompA* gene expression in mid-log phase *E. cloacae* was significantly reduced in the PBS-G condition when compared to the mock control (no virus), which is consistent with previous research revealing that *ompA* expression within *E. coli* grown to log phase is reduced when bacteria are grown in a glucose medium [33]. These results reveal that glucose likely has an impact on bacterial gene expression following MNV binding, however, they do not nullify the idea that viral attachment may be directly affecting bacterial gene expression independent of growth medium.

While examining the impact of glucose on stationary phase bacterial gene expression, although not significant, we found lower expression of *ompX*, *flgB*, *ftsL*, and *rcsC* in stationary phase M9 conditions compared to PBS diluent conditions. Conversely, *flgB* expression in the PBS diluent condition was significantly greater than expression in M9 media and all PBS-G media. Gene expression trends in all PBS-G were very similar to what was observed in M9 for all tested conditions when compared to the mock condition indicating that glucose is likely responsible for the gene expression changes observed in these media compared to PBS diluent condition. To test if *flgB* expression varies based on glucose concentration, we tested gene expression in stationary phase *E. cloacae* following MNV attachment in three different PBS-G conditions with varying glucose concentrations. Changing the glucose levels in PBS-G media did not significantly impact *flgB* expression, given the similar levels of expression observed among all mediums with glucose present when compared to the mock control. However, given that all resulted in significant decreases in glucose expression, it is still evident that glucose may be reducing *flgB* expression following MNV attachment. Given that there are no observed differences compared to the mock (no MNV) control, these data indicate that the changes in *flgB* expression are induced by the presence of glucose and not the virus. Previous studies on *E. coli* have revealed that glucose can have specific impacts on the gene expression of bacteria grown in its presence [15,22,34,35]. Previous data found that glucose reduced *ompX* expression in *E. coli* grown to stationary phase [15], which is consistent with our results. Furthermore, it has been previously shown that *ompX* expression levels reduce *E. coli* in minimal media compared to a PBS medium [23], and it has also been found that biofilm formation by *E. coli* is significantly lower in minimal media compared to PBS [14]. Both *ompX* and *rcsC* are involved in biofilm formation. Therefore, although no significant differences in gene expression were observed in *ompX* and *rcsC* following MNV attachment to stationary phase *E. cloacae*, it is probable that glucose could be the cause of the lower gene expression levels in the M9 and PBS-G conditions due to the higher gene expression levels in the PBS condition relative to the mock control. Minimal media with glucose has also been found to reduce flagellar motility, biofilm formation, and adhesion [23] as well as flagellar synthesis, which involves *flgB* [17,35]. Thus, it seems likely that the significant increase in *flgB* expression observed in the PBS condition and the lack of significant *flgB* expression change in the M9 and PBS-G conditions, when compared to the mock in stationary phase *E. cloacae* during MNV attachment, is impacted by the presence of glucose in the diluent medium, with glucose hindering the ability of MNV to increase *flgB* expression. We also examined changes in the expression of these bacterial genes during HNoV binding. Unlike MNV, HNoV did not significantly alter the expression of the targeted genes, however, the trends in gene expression were, in some cases, similar to MNV and were also consistent with previous RNA-seq analysis [12]. The observed differential impacts on gene expression between these viruses may be connected to the differing methods by which each type of norovirus attaches to the bacteria. A previous study has shown that MNV primarily attaches to the bacterial outer membrane [20], while HNoV displays more extensive binding to both the outer membrane and bacterial appendages [1,9]. Furthermore, previous work demonstrated that, while both viruses induce genome-wide changes in bacterial gene expression, they only share about one-third of the differentially regulated genes in common, indicating that these viruses alter bacterial gene expression in different manners [12]. In addition to binding proteinaceous structures like the outer membrane and flagella, HNoVs have been shown to bind prolifically to carbohydrate-based structures (i.e., HGBAs) on the bacterial cell surface [1], and it may be that this virus more significantly alters the expression of genes associated with the production of carbohydrate-based structure.

Ultimately, our data revealed that the expression of some target genes are altered in each tested condition following norovirus attachment and indicates that glucose plays a role in bacterial binding by MNV, which suggests that changes in gene expression alter the efficacy of viral binding to *E. cloacae*. MNV utilizes terminal sialic acids on the gangliosides of macrophages [36] and some mammalian commensal bacteria have been assumed to express sialic acid sugars within their capsular polysaccharides as a potential form of host mimicry [33,37]. This has led to speculation that MNV may also bind sialic acids on the bacterial cell surface [12]. Further research is needed to define the role of glucose in MNV attachment to commensal bacteria.

## 5. Conclusions

Our results reveal that the presence of glucose in minimal media has an impact on both murine norovirus attachment levels to *E. cloacae* as well as *E. cloacae* gene expression. Reduced MNV binding to *E. cloacae* occurred in the presence of a glucose medium. Additionally, the combination of MNV and glucose reduced the expression of surface structures gene previously associated with norovirus attachment. These changes in viral binding and gene expression due to glucose were also found to occur in a growth phase-dependent manner. Collectively, these data demonstrate that both growth phase and nutrient availability alter viral interactions with commensal bacteria, potentially due to changed expression of the structures to which norovirus binds. Future studies will determine the precise role of glucose in norovirus attachment and how the impact of nutrients may alter the impact of commensal bacteria on murine norovirus infection in vivo. Our research ultimately advances our understanding of norovirus–bacterium interactions and will lead to further studies regarding how enteric viruses and commensal bacteria interact, potentially leading to a better understanding of norovirus pathogenesis.

## Figures and Tables

**Table 1:** The five target genes and their functions.

Target Gene	Protein Name	Function
*ompA*	Outer membrane protein A	Outer membrane protein; receptor, adhesin, and biofilm formation
*ompX*	Outer membrane protein X	Outer membrane protein; adhesion, cell defense
*flgB*	Flagellar basal body rod protein	Structural component of flagellum
*ftsL*	Cell division protein	Role in cell division
*rcsC*	Sensor histidine kinase	Sensor kinase; biofilm formation

## Data Availability

The data presented in this study are available upon request to the corresponding author.

## Supplementary Materials

The following supporting information can be downloaded at: https://www.mdpi.com/article/10.3390/v14081596/s1, Table S1: Primers used for qPCR. Figure S1: Comparison of *flgB* expression with differing levels of glucose.

## References

[1] Almand E.A., Moore M.D., Outlaw J., Jaykus L.-A. (2017). Human norovirus binding to select bacteria representative of the human gut microbiota. PLoS ONE.

[2] Riddle M.S., Walker R.I. (2016). Status of vaccine research and development for norovirus. Vaccine.

[3] De Graaf M., Van Beek J., Koopmans M. (2016). Human norovirus transmission and evolution in a changing world. Nat. Rev. Genet..

[4] Green K.Y. (2014). Norovirus infection in immunocompromised hosts. Clin. Microbiol. Infect..

[5] Huang P., Farkas T., Marionneau S., Zhong W., Ruvoën-Clouet N., Morrow A.L., Altaye M., Pickering L.K., Newburg D.S., Le Pendu J. (2003). Noroviruses Bind to Human ABO, Lewis, and Secretor Histo–Blood Group Antigens: Identification of 4 Distinct Strain-Specific Patterns. J. Infect. Dis..

[6] Marionneau S., Ruvoën N., Le Moullac–Vaidye B., Clement M., Cailleau–Thomas A., Ruiz–Palacois G., Huang P., Jiang X., Le Pendu J. (2002). Norwalk virus binds to histo-blood group antigens present on gastroduodenal epithelial cells of secretor individuals. Gastroenterology.

[7] Jones M.K., Watanabe M., Zhu S., Graves C.L., Keyes L.R., Grau K.R., Gonzalez-Hernandez M.B., Iovine N.M., Wobus C.E., Vinjé J. (2014). Enteric bacteria promote human and mouse norovirus infection of B cells. Science.

[8] Springer G.F., Williamson P., Brandes W.C. (1961). Blood group activity of gram-negative bacteria. J. Exp. Med..

[9] Miura T., Sano D., Suenaga A., Yoshimura T., Fuzawa M., Nakagomi T., Nakagomi O., Okabe S. (2013). Histo-Blood Group Antigen-Like Substances of Human Enteric Bacteria as Specific Adsorbents for Human Noroviruses. J. Virol..

[10] Li D., Breiman A., Le Pendu J., Uyttendaele M. (2015). Binding to histo-blood group antigen-expressing bacteria protects human norovirus from acute heat stress. Front. Microbiol..

[11] Baldridge M.T., Nice T.J., McCune B.T., Yokoyama C.C., Kambal A., Wheadon M., Diamond M.S., Ivanova Y., Artyomov M., Virgin H.W. (2015). Commensal microbes and interferon-&lambda; determine persistence of enteric murine norovirus infection. Science.

[12] Mosby C.A., Bhar S., Phillips M.B., Edelmann M.J., Jones M.K. (2022). Interaction with mammalian enteric viruses alters outer membrane vesicle production and content by commensal bacteria. J. Extracell. Vesicles.

[13] Ishihama A. (1999). Modulation of the nucleoid, the transcription apparatus, and the translation machinery in bacteria for stationary phase survival. Genes Cells.

[14] Bleotu C., Chifiriuc M.C., Mircioaga D., Sandulescu O., Aldea I.M., Banu O., Ion D., Diaconu C.D., Marinescu F., Lazar V. (2017). The influence of nutrient culture media on *Escherichia coli* adhesion and biofilm formation ability. Rom. Biotechnol. Lett..

[15] Yang J.-N., Wang C., Guo C., Peng X.-X., Li H. (2011). Outer membrane proteome and its regulation networks in response to glucose concentration changes in *Escherichia coli*. Mol. Biosyst..

[16] Zeraik A.E., Nitschke M. (2012). Influence of growth media and temperature on bacterial adhesion to polystyrene surfaces. Braz. Arch. Biol. Technol..

[17] Adler J., Templeton B. (1967). The Effect of Environmental Conditions on the Motility of Escherichia coli. J. Gen. Microbiol..

[18] Holst J.J., Gribble F., Horowitz M., Rayner C.K. (2016). Roles of the Gut in Glucose Homeostasis. Diabetes Care.

[19] Anhê F.F., Barra N.G., Schertzer J.D. (2020). Glucose alters the symbiotic relationships between gut microbiota and host physiology. Am. J. Physiol. Metab..

[20] Madrigal J.L., Bhar S., Hackett S., Engelken H., Joseph R., Keyhani N.O., Jones M.K. (2020). Attach Me If You Can: Murine Norovirus Binds to Commensal Bacteria and Fungi. Viruses.

[21] Livak K.J., Schmittgen T.D. (2001). Analysis of relative gene expression data using real-time quantitative PCR and the 2^−ΔΔCT^ Method. Methods.

[22] Lin H.-H., Hsu C.-C., Yang C.-D., Ju Y.-W., Chen Y.-P., Tseng C.-P. (2011). Negative Effect of Glucose on *ompA* mRNA Stability: A Potential Role of Cyclic AMP in the Repression of *hfq* in *Escherichia coli*. J. Bacteriol..

[23] Li B., Huang Q., Cui A., Liu X., Hou B., Zhang L., Liu M., Meng X., Li S. (2018). Overexpression of Outer Membrane Protein X (OmpX) Compensates for the Effect of TolC Inactivation on Biofilm Formation and Curli Production in Extraintestinal Pathogenic *Escherichia coli* (ExPEC). Front. Cell. Infect. Microbiol..

[24] Rossi E., Paroni M., Landini P. (2018). Biofilm and motility in response to environmental and host-related signals in Gram negative opportunistic pathogens. J. Appl. Microbiol..

[25] Janderová B., Kapralek F., Julak J. (1980). Effect of glucose on the biochemical properties of the bacterial cytoplasmic membrane. Folia Microbiol..

[26] Zhu L., Liu X.-K., Zhao G., Zhi Y.-D., Bu X., Ying T.-Y., Feng E.-L., Wang J., Zhang X.-M., Huang P.-T. (2007). Dynamic Proteome Changes of Shigella flexneri 2a During Transition from Exponential Growth to Stationary Phase. Genom. Proteom. Bioinform..

[27] Naves P., del Prado G., Huelves L., Gracia M., Ruiz V., Blanco J., Rodríguez-Cerrato V., Ponte M., Soriano F. (2008). Measurement of biofilm formation by clinical isolates of *Escherichia coli*is method-dependent. J. Appl. Microbiol..

[28] Tao H., Bausch C., Richmond C., Blattner F.R., Conway T. (1999). Functional Genomics: Expression Analysis of *Escherichia coli* Growing on Minimal and Rich Media. J. Bacteriol..

[29] Kubota H., Senda S., Nomura N., Tokuda H., Uchiyama H. (2008). Biofilm Formation by Lactic Acid Bacteria and Resistance to Environmental Stress. J. Biosci. Bioeng..

[30] Makinoshima H., Aizawa S.-I., Hayashi H., Miki T., Nishimura A., Ishihama A. (2003). Growth Phase-Coupled Alterations in Cell Structure and Function of Escherichia coli. J. Bacteriol..

[31] Mañas P., Mackey B.M. (2004). Morphological and Physiological Changes Induced by High Hydrostatic Pressure in Exponential- and Stationary-Phase Cells of *Escherichia coli*: Relationship with Cell Death. Appl. Environ. Microbiol..

[32] Ma Q., Wood T.K. (2009). OmpA influences *Escherichia coli* biofilm formation by repressing cellulose production through the CpxRA two-component system. Environ. Microbiol..

[33] Lewis A.L., Desa N., Hansen E.E., Knirel Y.A., Gordon J.I., Gagneux P., Nizet V., Varki A. (2009). Innovations in host and microbial sialic acid biosynthesis revealed by phylogenomic prediction of nonulosonic acid structure. Proc. Natl. Acad. Sci. USA.

[34] Park S., Park Y.-H., Lee C.-R., Kim Y.-R., Seok Y.-J. (2016). Glucose induces delocalization of a flagellar biosynthesis protein from the flagellated pole. Mol. Microbiol..

[35] El-Kazzaz W., Morita T., Tagami H., Inada T., Aiba H. (2004). Metabolic block at early stages of the glycolytic pathway activates the Rcs phosphorelay system via increased synthesis of dTDP-glucose in *Escherichia coli*. Mol. Microbiol..

[36] Taube S., Perry J.W., Yetming K., Patel S.P., Auble H., Shu L., Nawar H.F., Lee C.H., Connell T.D., Shayman J.A. (2009). Ganglioside-Linked Terminal Sialic Acid Moieties on Murine Macrophages Function as Attachment Receptors for Murine Noroviruses. J. Virol..

[37] Carlin A.F., Uchiyama S., Chang Y.-C., Lewis A.L., Nizet V., Varki A. (2009). Molecular mimicry of host sialylated glycans allows a bacterial pathogen to engage neutrophil Siglec-9 and dampen the innate immune response. Blood.

