# Supplementary material for: Glucose Reduces Norovirus Binding to Enterobacter cloacae and Alters Gene Expression of Bacterial Surface Structures in a Growth Phase Dependent Manner
Source: Viruses. 2022 Jul 22;14(8):1596. doi: 10.3390/v14081596 (PMC9331879; doi:10.3390/v14081596)
Supplement: Supplementary file 1 [file viruses-14-01596-s001.zip › viruses-1700602-supplementary.pdf]

Table S1. Primers used for qPCR.

Gene	Sequence (5'->3')
Enterobacter cloacae primers	
rpoB-Fwd	TACGCGTCGACCCAACCAAC
rpoB-Rev	TCGATCTCATCGCGCAGCAG
flgB-Fwd	GCGCCCACGACCGATTTACT
flgB-Rev	ACGCTCCCGGTCCATATCCA
ftsL-Fwd	TAGGAAGCAACGAGCGCCAT
ftsL-Rev	GCAGAGTGGCAGCTTCCCAA
ompA -Fwd	GCAGCGGTGACAACATCATC
ompA -Rev	ATCACATTCACGGCGGTCTT
ompX-Fwd	ACGACTGGGCAAGCATCTAC
ompX-Rev	GAGAAGCCGTAGTCGCTGTT
rcsC-Fwd	GGCTGGCAATTTGTGAGAAG
rcsC-Rev	GCGCAGAATAGAGCGGAATA
Murine Norovirus Primers	
MNV-1 Fwd	GTGCGCAACACAGAGAAACG
MNV-1 Rev	CGGGCTGAGCTTCCTGC

Figure S1. Comparison of *flgB* expression with differing levels of glucose.

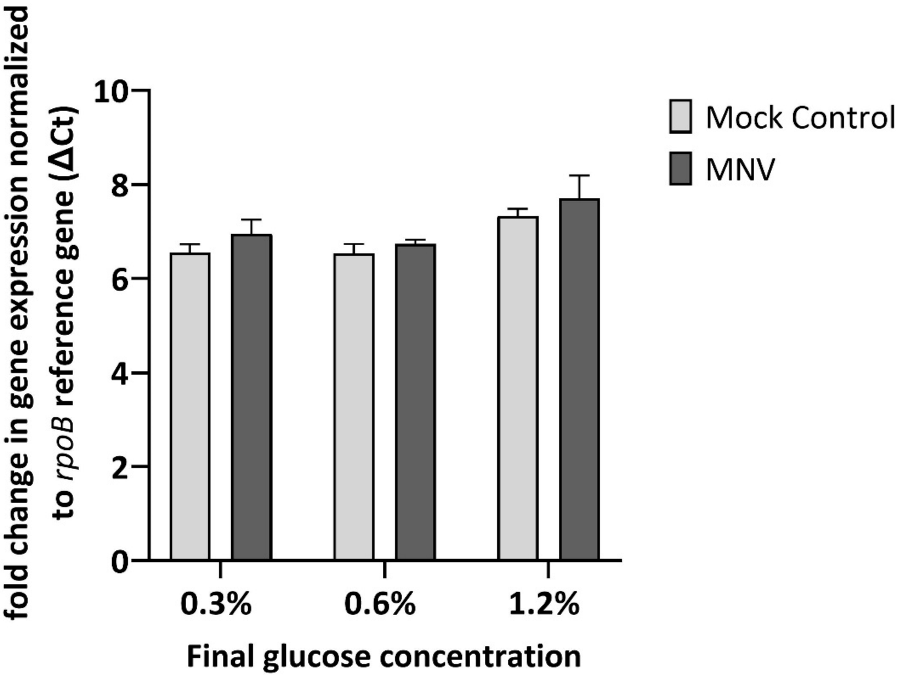

Values shown illustrate the fold change in gene expression of *flgB* normalized to *rpoB* reference gene using the ΔCt method. ($Ct_{(gene\ of\ interest)}$ - $Ct_{(reference\ gene)}$). The final concentration of glucose in PBS is shown on the x-axis. All comparisons were non-significant with p-values of > 0.05. Error bars are SEM with n = 3 biological replicates.
